# Late cornified envelope 1C (LCE1C), a transcriptional target of TAp63 phosphorylated at T46/T281, interacts with PRMT5

**DOI:** 10.1038/s41598-018-23045-7

**Published:** 2018-03-20

**Authors:** Norikazu Yabuta, Chie Ota, Towa Sasakura, Yoko Naito, Daisuke Okuzaki, Kohshiro Fukushima, Hiroshi Nojima

**Affiliations:** 1 Department of Molecular Genetics, Research Institute for Microbial Diseases, Osaka University, 3-1 Yamadaoka, Suita, Osaka 565-0871 Japan; 2 DNA-chip Development Center for Infectious Diseases, Research Institute for Microbial Diseases Osaka University, 3-1 Yamadaoka, Suita, Osaka 565-0871 Japan

## Abstract

p63, a transcriptional factor that belongs to the p53 family, regulates epidermal differentiation, stemness, cell death, tumorigenesis, metastasis, and senescence. However, its molecular mechanism remains elusive. We report here that TAp63 phosphorylated at T46/T281 specifically upregulates the late cornified envelope 1C (*LCE1C*) gene that is essential at a relatively late stage of epithelial development. We identified these phosphorylation sites during a search for the targets of Cyclin G-associated kinase (GAK) *in vitro*. *LCE1C* was drastically upregulated by doxycycline-dependent expression of Myc-TAp63 wild-type protein. Luciferase reporter assays using the promoter region of the *LCE1C* gene confirmed that the phosphorylations of TAp63-T46/T281 contributed to full transcriptional activation of the *LCE1C* gene. LCE1C interacted with protein arginine methyltransferase 5 (PRMT5) and translocated it from the nucleus to the cytoplasm. Mass spectrometry and co-immunoprecipitation identified importin-α as one of the association partners of LCE1C. In summary, we propose that the GAK_TAp63-pT46/pT281_LCE1C axis plays an important role in preventing the nuclear function of PRMT5.

## Introduction

As a member of the p53/p63/p73 family, p63 is a transcriptional regulator that is involved in epidermal differentiation, stemness, cell death, tumorigenesis, metastasis, and senescence^1^. p63 can individually induce cell cycle arrest and also collaborates with other members, including p53, p73 and other splicing isoforms (for example, γ isoform) of p63, to induce apoptosis in response to DNA damage^2–4^. Notably, p63 exists as a complex gene that encodes multiple isoforms by alternative splicing, which can be classified into two categories: isoforms with an acidic transactivation domain (TA isoforms); and isoforms that lack this domain (DN isoforms). TAp63 (TA) isoform that contains the N-terminal transactivation domain possesses tumor-suppressive activities and activates apoptosis and suppresses metastasis^5^. By contrast, ΔNp63 (DN) isoform lacking the N-terminal transactivation domain of p63 is commonly expressed and acts as a dominant negative inhibitor of p63 function with gain of oncogenic properties^6^. Although it is traditionally accepted that TAp63 has tumor-suppressive activities while ΔNp63 has oncogenic activities, this model does not necessarily apply to all functions of p63 in cancer and normal tissue developments. For example, ΔNp63 can transcriptionally induce caspase-1 to suppress tumorigenesis and MKP3 to inhibit metastasis^7,8^. Moreover, ΔNp63 is primarily expressed in the normal epidermis (to be involved in epithelial development) or in head and neck squamous cell carcinomas and non-small cell lung cancers, whereas TAp63 is primarily expressed in oocytes or in malignant lymphomas^1^. The physiological function of p63 in normal tissue development and tumorigenesis is further complicated by multiple isoforms of p63 and a correlation with other p53 family. Therefore, understanding of its molecular mechanism is still limited.

The cornified cell envelope (CE), an insoluble protein-lipid matrix, substitutes the plasma membrane in terminally differentiating keratinocytes to function as an epidermal water barrier^9^. Most of the CE genes are clustered on human chromosome one (mouse chromosome three) and encode homologous stratum-corneum proteins, suggesting their evolution by duplication of a common ancestral gene when life acquired the ability to survive on land, since no CE components are found in fish and amphibians^10^. Late cornified envelope group I (LCE1) genes reside in this cluster and are expressed at a relatively late stage of epithelial development in mouse, being integrated into the CE through transglutaminase-mediated cross-linking in the process of envelope maturation^11^. LCE genes respond to environmental stimuli such as calcium levels and ultraviolet (UV) light, but the biological significance of this remains elusive. Notably, LCE1 genes are downstream targets of p53^12^ and have tumor suppressor functions through modulating the activity of protein arginine methyltransferase 5 (PRMT5).

Cyclin G-associated kinase (GAK), an association partner of clathrin heavy chain, localizes to both the cytoplasm and nucleus with distinct association modes^13^. In the cytoplasm, GAK plays an essential role in membrane trafficking as a cofactor for the HSC70-dependent uncoating of clathrin-coated vesicles^14^. In the nucleus, GAK acts as a transcriptional coactivator of the androgen receptor^15^. Indeed, GAK expression level is positively correlated with malignancy in surgical specimens from prostate cancer patients^16^. GAK also acts as a regulator of proper cell cycle progression during M phase because knockdown of GAK using small interfering RNAs (siRNAs) results in cell cycle arrest at metaphase, causing abnormal centrosome and chromosome stability^17^. GAK knockout (GAK^−/−^) mice display embryonic lethality^18^ and mouse embryonic fibroblasts (MEFs) derived from GAK^−/−^ mice fail to divide and ultimately become senescent^19^, suggesting an essential role for GAK in cell growth. Osteosarcoma cells of patients with a poor prognosis display increased expression of GAK in the nucleus, and siRNA-mediated knockdown of GAK decreases the propagation of osteosarcoma cells^20^. Thus, novel kinase targets of GAK in the nucleus are expected to be identified to elucidate the role of GAK in cancer cells.

Here, we show that GAK phosphorylates TAp63 at T46 and T281 *in vitro*. DNA microarray analysis identified *LCE1C* as a novel transcriptional target of TAp63, and the mRNA level of *LCE1C* was higher in TAp63-expressing cells than in empty vector-expressing cells. Luciferase reporter assays using the promoter region of the *LCE1C* gene confirmed that transcription of *LCE1C* was inhibited by the expression of non-phosphorylatable mutant cells. Western blot analysis (Wb) revealed that LCE1C proteins tend to form oligomers, and dimerized LCE1C preferentially associated with PRMT5. We propose that the GAK_TAp63-pT46/pT281_LCE1C axis constitutes a novel function of TAp63, partially explaining the phenotype of p63-null mice, and this axis may contribute to suppression of the tumorigenesis through PRMT5.

## Results

### TAp63 is a phosphorylation target of GAK *in vitro*

**Figure 1:**
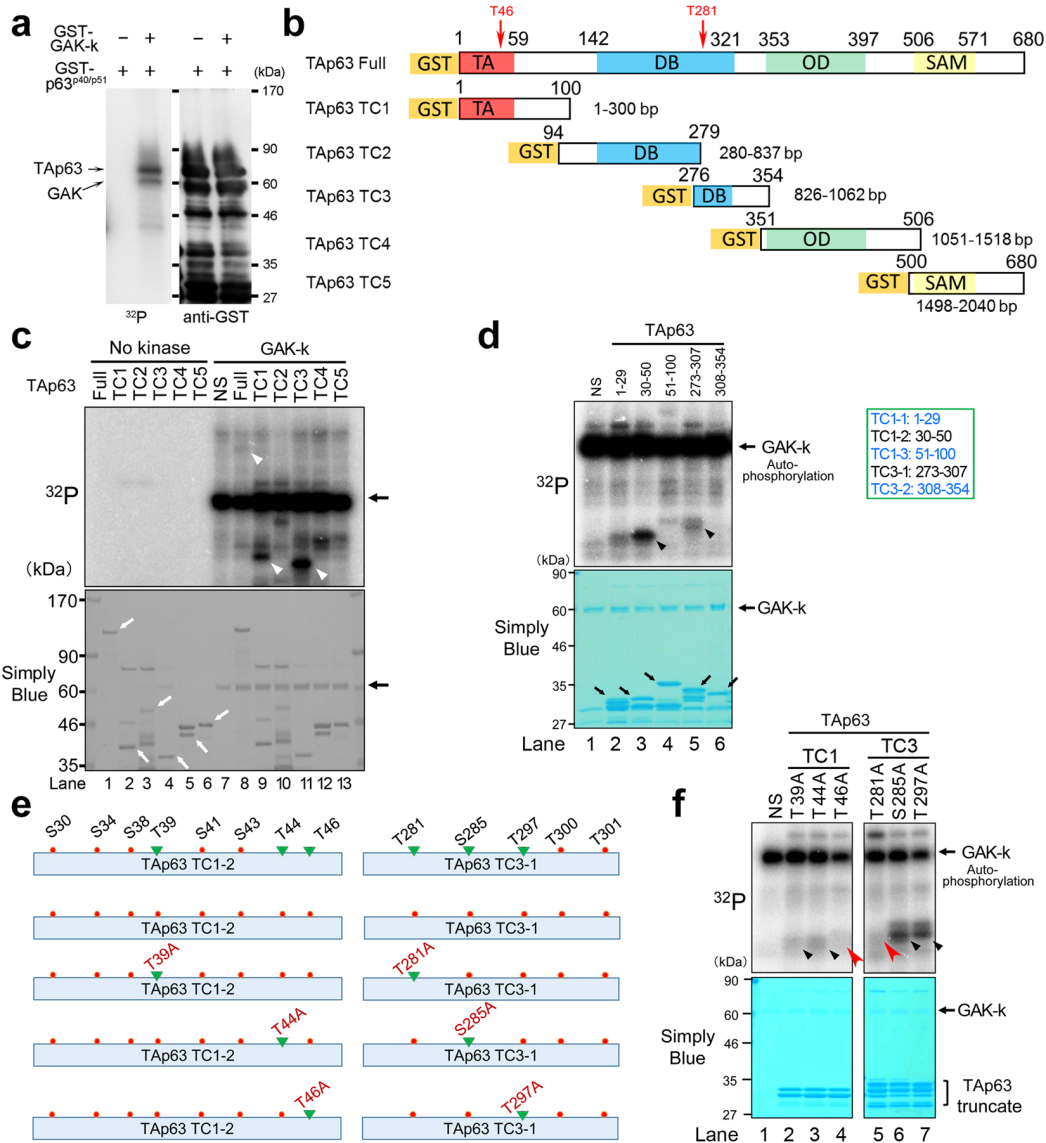
TAp63 is phosphorylated at T46 and T281 by GAK *in vitro*. (**a**) GST-p63^p40/p51^ protein shows a phosphorylated band (horizontal arrow) in the presence of GST-tagged GAK-k (tilted arrow), the kinase domain of GAK. (**b**) A schematic representation of TAp63 structure and TC1–TC5 fragments denoting the sites of functional domains and amino acid numbers. TA, transactivation; DB, DNA binding; OD, oligomerization; SAM, C-terminal sterile alpha motif. (**c**) Radio-autograph of SDS-PAGE analysis after *in vitro* kinase assays of full-size and TC1–TC5 fragments of TAp63 in the absence (no kinase) or presence of GAK-k (horizontal arrow). White arrows denote the bands for full-size and TC1–TC5 fragments of TAp63 as stained by Simply Blue, whereas white arrowheads indicate the phosphorylated bands in the radio-autograph. Horizontal arrows denote the band for auto-phosphorylation of GAK-k. (**d**) Radio-autograph showing that TC1-2 (30–50) and TC3-1 (273–307) fragments, but not TC1-1 (1–29), TC1-3 (51–100), or TC3-2 (306–354) fragments, of TAp63 are the phosphorylation substrates of GAK-k. Black arrowheads or black arrows denote the bands for phosphorylated TAp63 fragments or GAK-k, respectively. NS, no substrate. (**e**) A schematic representation of TC1-2 and TC3-1 fragments of TAp63 that were identified as the phosphorylated substrates by GAK-k. Location of S and T residues that are possible phosphorylation targets of GAK-k are shown, together with their alanine (A) substitutes that are used as the GST-tagged substrates (green triangles). (**f**) Radio-autograph showing that the band intensities for T46A and T281A fragments were weakened (red arrowheads) due to T/A substitutions. Black arrowheads denote the phosphorylated bands for TC1-T39A, TC1-T44A, TC3-285A, and TC3-297A fragments. Horizontal black arrows denote the bands for GAK-k.

During the search for phosphorylation targets of GAK in the nucleus, we identified TAp63 as a substrate for phosphorylation *in vitro* (Fig. 1a). When we dissected TAp63 into five fragments and prepared their affinity purified GST-tagged proteins (Fig. 1b), TC1 (1–100) and TC3 (276–354) fragments were phosphorylated by GAK more strongly than full-size TAp63 protein (white arrowheads in Fig. 1c). We next divided TC1 and TC3 fragments into three (1–29, 30–50, and 51–100) or two (273–307 and 308–354) portions and found that TC1–2 (30–50) and TC3–1 (273–307) portions were phosphorylated conspicuously (black arrowheads in Fig. 1d). Because GAK preferably phosphorylates threonine (T), we prepared five kinds of affinity purified GST-tagged mutant proteins in which the indicated T (green triangle in Fig. 1e) was replaced by alanine (A) to abolish phosphorylation at these sites (T39A, T44A, T46A, T281A, and T297A). We also prepared S285A mutant as an example of a serine (S) candidate. We found that T39A and T44A mutant proteins showed weakly, whereas T285A and T297A mutant proteins showed strongly, phosphorylated bands (black arrowheads in Fig. 1f). By contrast, T46A and T281A mutant proteins showed only faint phosphorylated bands (red arrowheads in Fig. 1f). Taken together, we conclude that GAK phosphorylates TAp63 at T46 and T281 in the transactivation and DNA binding domains, respectively (red arrows in Fig. 1b).

### Identification of the transcriptional target of TAp63

**Figure 2:**
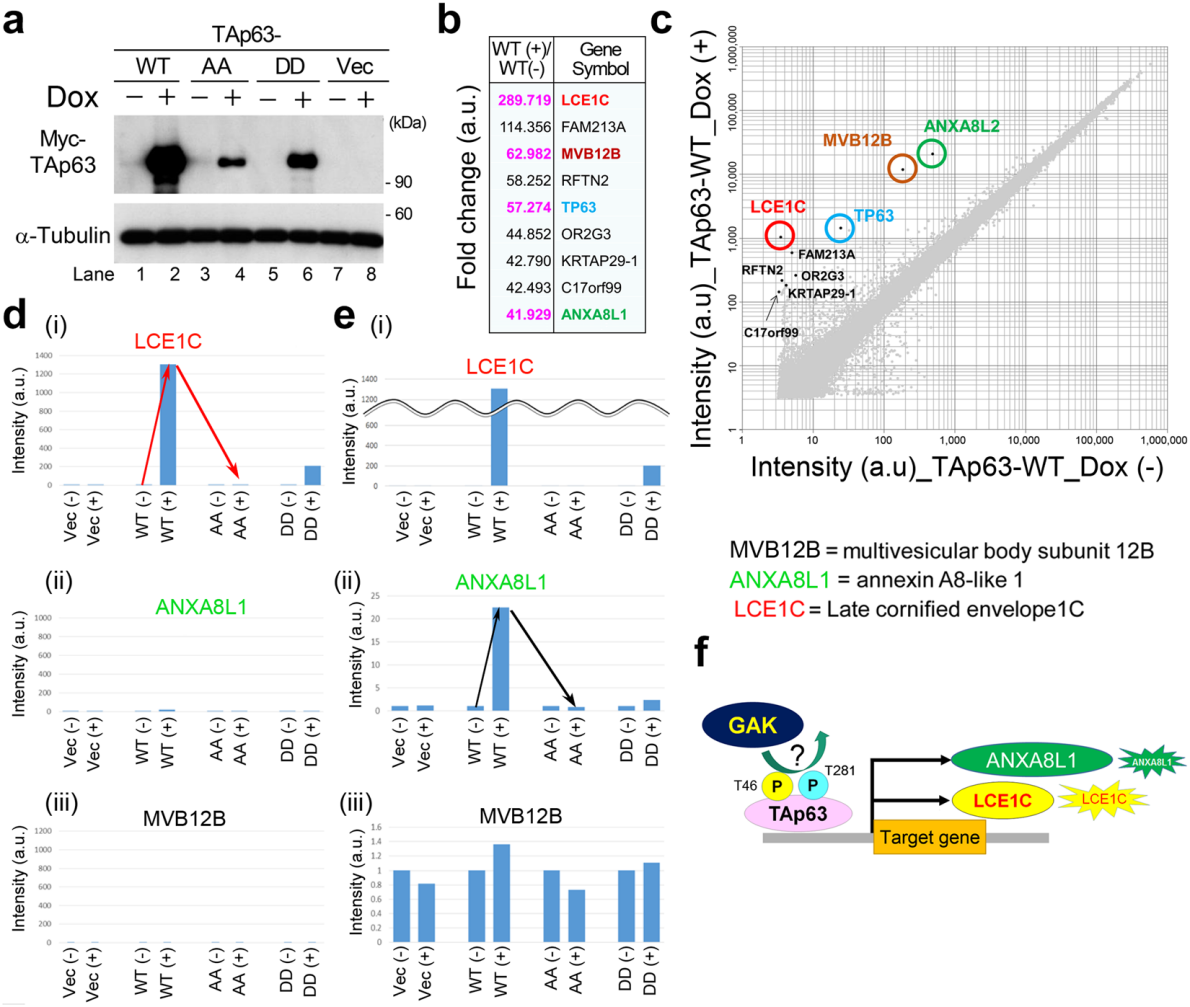
LCE1C is a transcriptional target of TAp63. (**a**) Wb shows that expression of Myc-TAp63 proteins drastically increased from Dox (−) to Dox (+) conditions in Tet-ON inducible Myc-TAp63-WT, Myc-TAp63-T46A/T281A (AA), or Myc-TAp63-T46D/T281D (DD) U2OS cell lines. Vec means Myc-vector alone. The amount of α-tubulin was monitored as a loading control. (**b**) List of the nine genes arranged in the descending order of averaged fold change values of signal intensity, obtained by DNA microarray analysis. (**c**) Scatter plot of the DNA microarray data for average values from Dox (−) versus Dox (+) conditions in Tet-ON inducible Myc-TAp63-WT cells. The y-axis and x-axis show log_2_[hybridization signal intensity] of microarray data from Dox (+) and Dox (−), respectively. Notable genes with high fold change values are plotted. (**d,e**) Bar graphs of qRT-PCR data show relative mRNA levels in Tet-ON inducible Myc-vector, Myc-TAp63-WT, Myc-TAp63-T46A/T281A (AA), or Myc-TAp63-T46D/T281D (DD) U2OS cell lines in Dox (−) and Dox (+) conditions for the denoted genes (i–iii). Red and black arrows highlight the notable alterations of the mRNA levels. The y-axis values are magnified in (e). (**f**) A schematic representation of the GAK_TAp63-T46/T281_LCE1C axis.

Because TAp63 is a transcription factor, we next examined if TAp63-T46/T281 phosphorylation might alter the expression levels of its target genes using genome-wide cDNA microarray analyses. For this purpose, we generated a Tet-ON inducible U2OS cell line expressing Myc-tagged WT TAp63 (Fig. 2a), and purified RNAs from Myc-TAp63-WT cells in the presence (+) or absence (−) of doxycycline (Dox). To determine whether the fold change values (Fig. 2b) in mRNA levels were physiologically significant, we generated a scatter plot for Dox (+) vs. Dox (−) (Fig. 2c). Because fold changes of genes with raw signal intensity >100 in at least in one of the samples are generally considered to be physiologically significant, we focused our subsequent analysis on three novel candidate genes for transcriptional targets of TAp63; late cornified envelope 1C (*LCE1C*), multivesicular body subunit 12B (*MVB12B*), and annexin A8-like 1 (*ANXA8L1*) (Supplementary Table S1).

To confirm that the microarray signals accurately reflected mRNA levels in these cells, we performed quantitative reverse transcription-polymerase chain reaction (qRT-PCR) on these three genes. For this purpose, we also generated Tet-ON inducible cell lines expressing non-phosphorylated or constitutively phosphorylated forms of TAp63 in which the T46/T281 residues were replaced by alanine (A) or aspartic acid (D), respectively. We purified RNAs from U2OS cells expressing Myc (vector alone), Myc-TAp63-WT, Myc-TAp63-T46A/T281A (AA), or Myc-TAp63-T46D/T281D (DD) and obtained qRT-PCR data that ride clean to the standard curves (Fig. S1). The mRNA level of *LCE1C* drastically increased from the Dox (−) to Dox (+) condition in the WT cells (upward red arrow in Fig. 2d–i) and slightly increased from the Dox (−) to Dox (+) condition in the DD cells (Fig. 2d–i, 2e–i), whereas no such increase was observed in the AA mutant (downward red arrow in Fig. 2d–i). Unexpectedly, the expression levels of TAp63-WT, AA, and DD in the cell lines were not even close to each other under Dox (+) condition (Fig. 2a), although it is not clear the reason why the expression levels of AA and DD were lower than that of WT. Possible explanations are, for example, the proteins of AA and DD might be more unstable than WT, or the ectopically expressed mutant TAp63 proteins might functionally interfere with the gene expression of the mutant TAp63. Therefore, these results suggest that the expression of *LCE1C* gene is apparently induced by TAp63 in a protein level-dependent manner, although it remains unclear whether the phosphorylations of T46/T281 is essential for or contribute to the TAp63-mediated expression of *LCE1C* gene in this experimental system (Fig. 2f). Induction of *ANXA8L1* transcription was also observed, although the induction level was lower than that of *LCE1C* (upward or downward black arrows in Fig. 2e-ii). By contrast, the mRNA level of *MVB12B* was almost unaltered (Fig. 2d-iii). Thus, we concentrated our further analysis on LCE1C.

### TAp63 regulates transcription of the LCE1C gene

**Figure 3:**
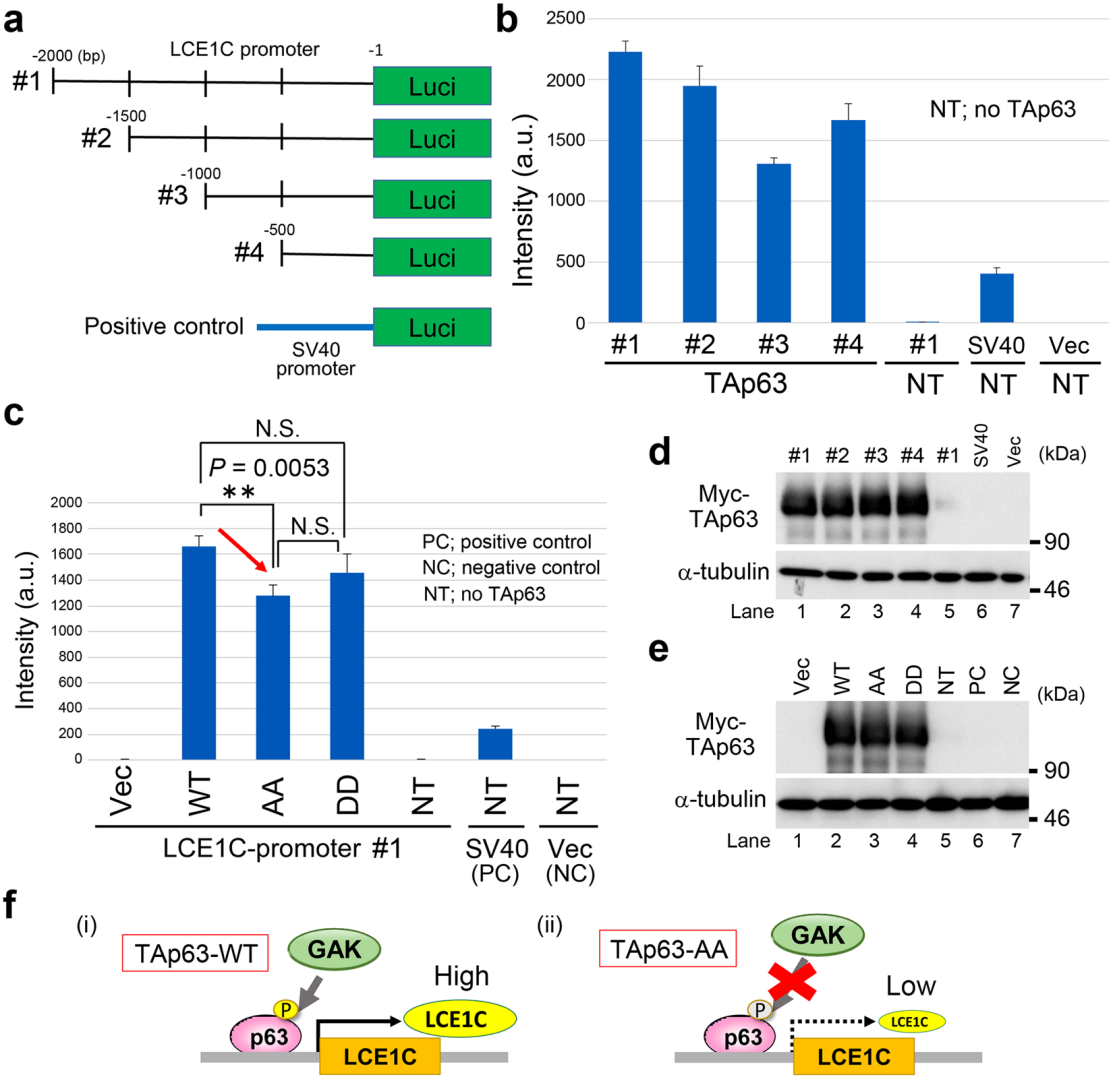
Luciferase reporter assays using the promoter region of the *LCE1C* gene. (**a**) A schematic representation of the promoter region (~2000 bp) of *LCE1C*. Green box indicates the location of the luciferase gene. (**b**) Bar graph to indicate the promoter activity of #1–4 promoter regions (arbitrary units). Promoter activity of SV40 promoter was measured as a positive control. Promoter activities using no TAp63 DNA (NT) or vector alone (Vec) in the reaction mixture were also measured as negative controls. (**c**) Bar graph to examine the alteration of the promoter activity of LCE1C-promoter #1 by addition of Myc-TAp63-WT, Myc-TAp63-T46A/T281A (AA), or Myc-TAp63-T46D/T281D (DD) plasmids into the reaction mixture. Promoter activities using no TAp63 DNA (NT) or vector alone (Vec) in the reaction mixture were also measured as negative controls (NC). Addition of SV40 promoter was examined as a positive control (PC). N.S. indicates no significant difference. (**d,e**) Wb to confirm the exogenous expression of Myc-TAp63 in luciferase assays. α-tubulin was detected as a loading control. (**f**) A schematic representation of the action of the GAK_TAp63-T46/T281_LCE1C axis (i), which was destroyed in the Myc-TAp63-T46A/T281A (AA) expressing cells (ii).

To examine if TAp63 or its phosphorylation regulates transcription of *LCE1C*, we performed luciferase reporter assays by systematically shaving the *LCE1C* promoter region (~2 kb) from the 5′ end by 500 bp each time, which harbors the putative p63 binding sites (Fig. S2), and linking each DNA fragment to the luciferase gene (Fig. 3a). Because the Tet-ON inducible cell lines expressing mutant TAp63 were not suitable for assessing the expression levels of *LCE1C* gene in these cells (Fig. 2a), TAp63-WT, AA, and DD mutants were transiently co-transfected with reporter gene constructs. TAp63 bound to this *LCE1C* promoter region and activated luciferase transcription, with promoter region #1 (~2 kb) giving the highest activation (Fig. 3b). Promoter activity of all four fragments was higher than that of the SV40 promoter (positive control), whereas almost no transcriptional activation was seen in the negative control (no TAp63) or with vector alone (Fig. 3b). Moreover, the level of this transcriptional activation was lower in the AA mutant, but it was partially restored in the DD mutant, further suggesting the importance of TAp63-T46/T281 phosphorylations for induction of *LCE1C* transcription, although the DD mutant might be unable to perfectly function as a phospho-mimic mutant (Fig. 3c). Wb confirmed successful expressions of Myc-TAp63 proteins in both luciferase assays (Fig. 3d, Fig. 3e). Taken together, we conclude that the transcription of *LCE1C* is induced by TAp63 of which full transcriptional activation might be supported by the phosphorylations of T46/T281 (Fig. 3f) because the expression of *LCE1C* was decreased in the AA mutant.

Next, to examine whether T281 of endogenous p63 is phosphorylated in the nucleus, we generated anti-TAp63-pT281 antibody, which was specific to phosphorylated form of TAp63-pT281 as judged by peptide dot blot (Fig. S3a). Wb using anti-p63 antibody detected several bands around the predicted size of endogenous p63 protein in HeLa S3 cells but little or no detectable levels in PC3 and U2OS cells (Fig. S3b), which is consistent with previous reports^21–23^. Because this phospho-specific antibody was not available for western blotting and U2OS cells had little or no expression of endogenous p63, the phosphorylation of endogenous TAp63-T281 was shown by immunofluorescence (IF) using HeLa S3 cells. IF indicated a nuclear localization of TAp63-pT281 signals, which disappeared when we performed peptide competition using phosphopeptide, but not non-phosphopeptide (Fig. S3c).

Furthermore, to confirm that the expression of *LCE1C* gene is promoted by TAp63 but not ΔNp63 in U2OS cells, we examined the expression level of endogenous ΔNp63 protein in Myc-TAp63-expressing U2OS cells and parental U2OS cells (Fig. S3d). Although human epidermoid carcinoma A431 cells had high expression of endogenous ΔNp63 protein (lanes 1 and 9)^24^, Myc-TAp63-expressing U2OS cells and parental U2OS cells had no expression of ΔNp63 protein (lanes 4 and 8). Thus, these results suggest that the expression of *LCE1C* gene is not influenced by ΔNp63 protein in U2OS cells.

### LCE1C interacts with PRMT5

**Figure 4:**
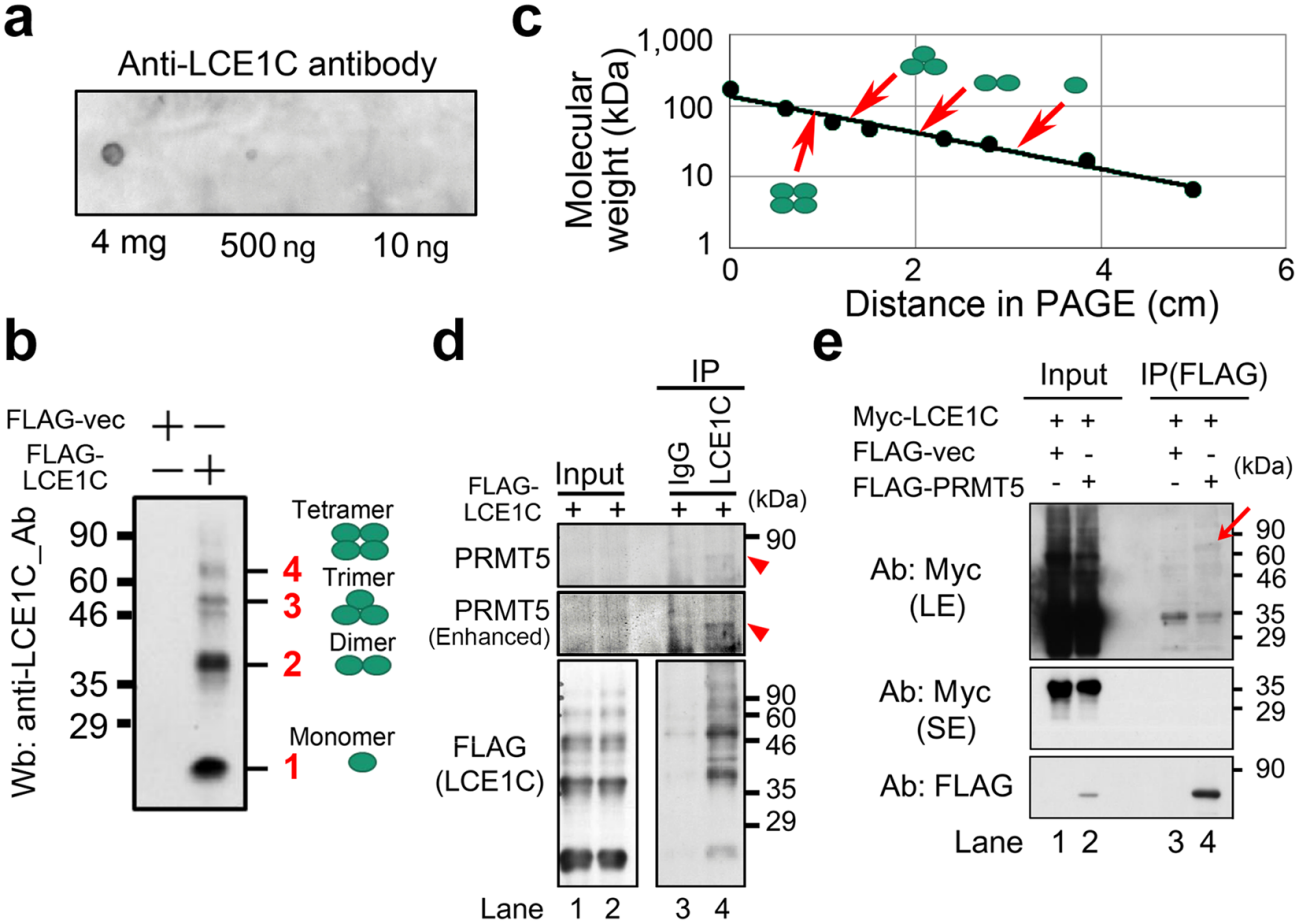
LCE1C is comprised of oligomers and interacts with PRMT5. (**a**) Dot blot analysis of the homemade anti-LCE1C antibody in which the denoted amount of antigen peptide (TPKCPPKCPTPKCPP) was dotted on the Wb filter. (**b**) Wb on the cell extracts from 293T cells expressing FLAG-vector alone or FLAG-LCE1C. Putative bands highlighted by red font (1, 2, 3, and 4) indicate monomer, dimer, trimer, or tetramer forms of LCE1C proteins. (**c**) A line graph shows a linear distribution of migration distance in SDS-PAGE used for Wb and molecular weight of LCE1C. Red arrowheads correspond to the band for indicated oligomers. (**d**) Co-IP on extract of 293T cells expressing FLAG-LCE1C shows an interaction of FLAG-LCE1C tetramer with PRMT5 (red arrowhead). (**e**) Co-IP on extract of 293T cells expressing Myc-LCE1C and/or FLAG-PRMT5 shows an interaction of FLAG-LCE1C tetramer with FLAG-PRMT5 (red arrow). LE; long exposure onto X-ray film. SE; short exposure onto X-ray film. Red arrow indicates a band for putative LCE1C tetramer.

LCE1F, a homolog of LCE1C, interacts with PRMT5 and regulates its activity to promote histone H3 methylation^12^. To examine if LCE1C also interacts with PRMT5, we first generated an anti-LCE1C antibody (Fig. 4a). Interestingly, LCE1C tended to form oligomers (Fig. 4b), as determined by a linear distribution of migration distance of the band in sodium dodecyl sulfate polyacrylamide gel electrophoresis (SDS-PAGE) (Fig. 4c). Co-immunoprecipitation (Co-IP) of the extract from U2OS cells revealed an interaction between PRMT5 and the LCE1C tetramer (red arrowhead in Fig. 4d). The weak band suggests that only a small population of these proteins interacted with each other or that the interaction itself was weak. Co-IP of 293T cells expressing both Myc-LCE1C and FLAG-PRMT5 also confirmed the interaction between FLAG-PRMT5 and Myc-LCE1C (Fig. 4e).

**Figure 5:**
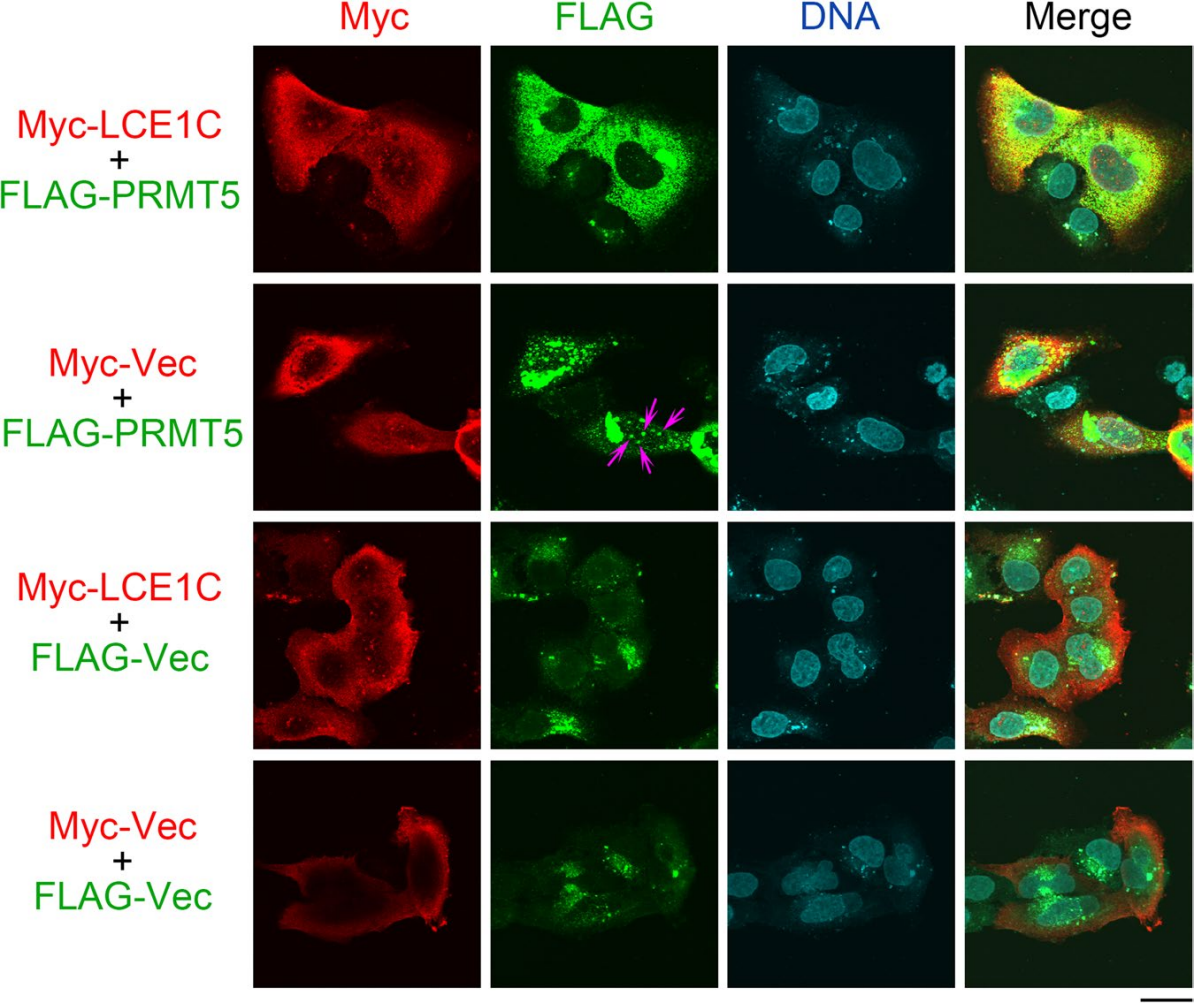
Subcellular localization of Myc-LCE1C and FLAG-PRMT5 in U2OS cells expressing these proteins. Pink arrows denote FLAG-PRMT5 signals showing nuclear dots. Scale bar, 30 µm.

A previous report showed that LCE1F and PRMT5 co-localize in both the nucleus and the cytoplasm^12^. Immunofluorescence (IF) images of U2OS cells co-expressing both Myc-LCE1C and FLAG-PRMT5 indicated that FLAG-PRMT5 localized predominantly to the cytoplasm (the top panels of Fig. 5). By contrast, FLAG-PRMT5 localized predominantly in the nucleus as dots in the absence of Myc-LCE1C when Myc-vec alone was co-expressed (pink arrows in the second row panel from top), suggesting a contribution of Myc-LCE1C for the cytoplasmic retention of FLAG-PRMT5. When we exchanged the FLAG and Myc tags, both FLAG-LCE1C and Myc-PRMT5 also showed cytoplasmic localizations (Fig. S4). Taken together, these results suggest that LCE1C promotes the cytoplasmic retention of PRMT5 or translocation of PRMT5 from the nucleus to the cytoplasm via the direct interaction, thereby preventing the nuclear function of PRMT5.

### LCE1C interacts with importin α

**Figure 6:**
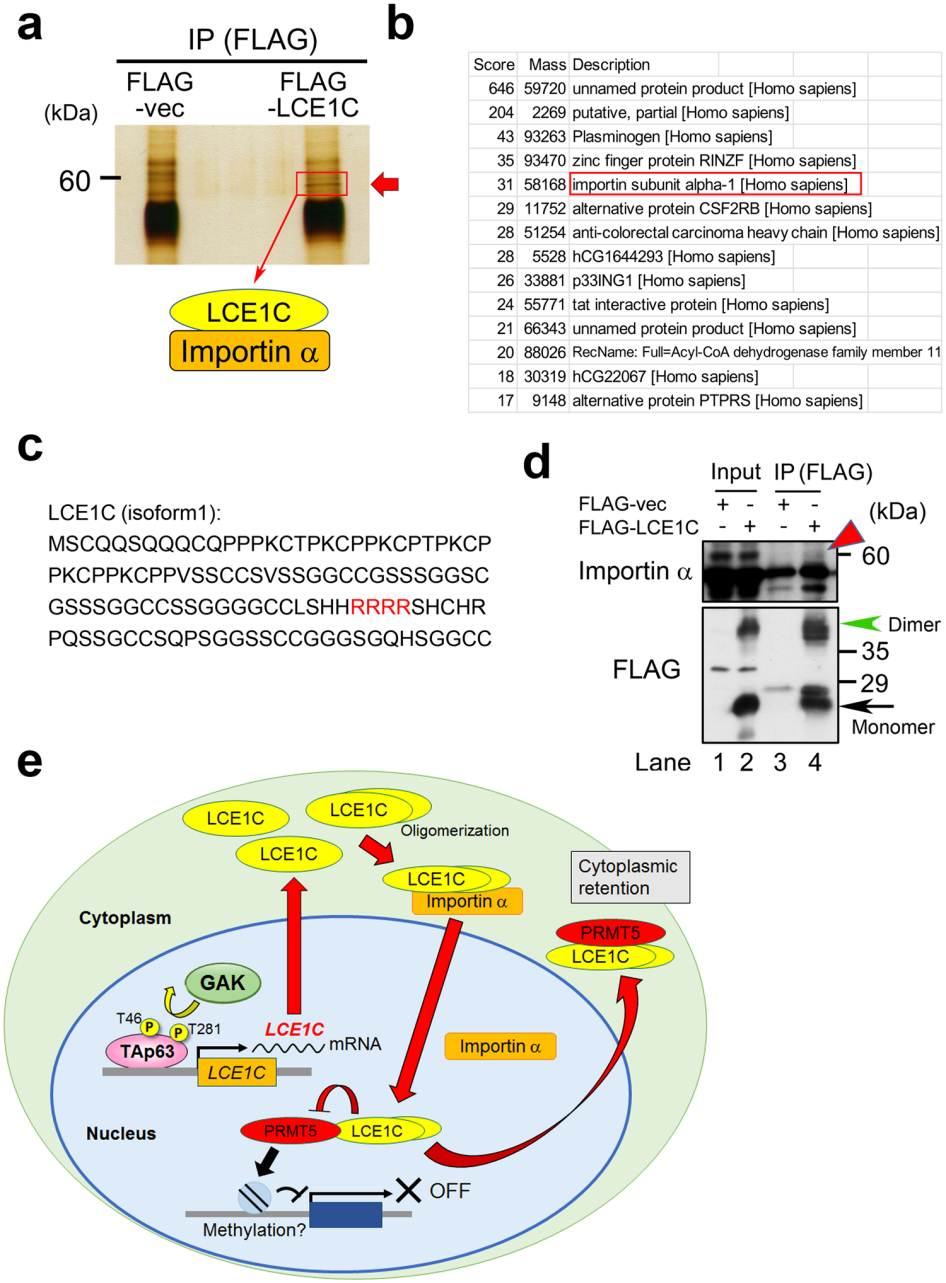
MS analysis identified importin α as one of the association partners of LCE1C. (**a**) Cell extracts prepared from logarithmically growing 293T cells expressing the FLAG-vector or FLAG-LCE1C were immunoprecipitated with an anti-FLAG antibody and the precipitates were applied to SDS-PAGE. After silver-staining, a strong band was detected only in 293T cells expressing FLAG-LCE1C; the gel fragment highlighted by a red rectangle was subjected to LC-MS/MS analysis. (**b**) A list of genes that were identified as association partners of LCE1C. Molecular weight and the score for each candidate protein are shown. The score signifies the “Mascot probability based scoring” that is used to judge whether a result is significant or not; the larger the number, the higher the probability that the detected peptide is derived from the denoted protein in the database. (**c**) Amino acid sequence of LCE1C protein. Candidate for NLS (RRRR) is highlighted by red font. (**d**) Co-IP analysis using the extract of 293T cells expressing the FLAG-vector or FLAG-LCE1C, which was immunoprecipitated with an anti-FLAG antibody. The precipitates were probed with anti-importin α or anti-FLAG antibodies. Red arrowhead indicates a band that shows an interaction between FLAG-LCE1C and importin α *in vivo*. Black arrow or green arrowhead indicates a band for monomer or dimer forms of FLAG-LCE1C proteins, respectively. (**e**) A schematic representation of the action of the GAK_TAp63-T46/T281_LCE1C axis, which is followed by interaction of LCE1C with importin α or PRMT5.

To further examine the physiological role of LCE1C, we searched for association partners of LCE1C using liquid chromatography-tandem mass spectrometry (LC-MS/MS). We transfected FLAG-vector and FLAG-LCE1C into U2OS cells, prepared cell extracts and performed IP using anti-FLAG antibody. After SDS-PAGE, we subjected the gel to silver-staining and detected a band (~60 kDa) that appeared only in FLAG-LCE1C-expressing cells (red rectangle and arrow in Fig. 6a). This band was analyzed by LC-MS/MS. Among the identified proteins with high scores (Fig. 6b), we noticed importin α because LCE1C harbors a putative nuclear localization signal (NLS) in the middle of the molecule (red font in Fig. 6c) and the predicted molecular weight of importin α was similar to the digested band size. This ~60 kDa band did not contain PRMT5 because the molecular weight of PRMT5 is predicted to be approximately 73 kDa. LCE1C might also be unable to simultaneously interact with both PRMT5 and importin α. Indeed, Co-IP showed the *in vivo* association of FLAG-LCE1C with importin α (red arrowhead in Fig. 6d). The weak band of importin α suggests that only a small percentage of these proteins interact with each other or that their interaction itself is weak. However, because immunofluorescence data showed that only a small portion of Myc-LCE1C or FLAG-LCE1C localized in the nucleus (Figs 5, S4), the subcellular localization of LCE1C might be hardly affected by the putative NLS. Moreover, because we could not find the nuclear export signal sequence (NES) in the ORF of LCE1C-isoform 1, the nucleocytoplasmic shuttling of LCE1C may require the interaction with other unknown factors. Notably, an almost equal amount of monomer (black arrow) and dimer (green arrowhead) forms of FLAG-LCE1C existed in this cell extract.

Taken together, our results suggest that TAp63 phosphorylated at T46 and T281 by GAK is fully activated and substantially upregulates the transcription of *LCE1C* genes. Thus, we proposed a model that excess LCE1C proteins readily form oligomers, including dimers, in the cytoplasm, which is transported into the nucleus via the interaction with importin α and other factor(s), and then LCE1C interacts with and translocates PRMT5 from the nucleus to the cytoplasm, thereby preventing the nuclear function of PRMT5, such as histone methylation (Fig. 6e). In the case of cancer cells with truncated mutant p63 proteins such as ΔNp63, the insufficient expression of LCE1C may be defective in inhibiting the oncogenic activity of PRMT5 because ΔNp63 harbors T281 in the DNA-binding domain but not T46 in the transactivation domain.

## Discussion

Here, we used DNA microarray and qRT-PCR analyses to identify *LCE1C* as a novel transcriptional target gene of TAp63 that is phosphorylated at T46 and T281 by GAK (Figs 1 and 2a–c). Notably, qRT-PCR revealed that the mRNA level of *LCE1C* in U2OS cells expressing TAp63-WT was dramatically increased in a Dox-dependent manner (Fig. 2d,e), suggesting that the transcription of *LCE1C* is dependent on the amount of TAp63 protein. Moreover, luciferase reporter assays using the promoter region of the *LCE1C* gene revealed that transcription of *LCE1C* was markedly induced in WT-expressing cells similarly to qRT-PCR, but the LCE1C promoter activity was apparently decreased in AA-expressing cells (Fig. 3). These results suggest that GAK contribute to the full activation of TAp63 for transcription of *LCE1C* through phosphorylation of TAp63-T46/T281 (Fig. 2f). Because T46 and T281 reside in the transactivation domain and DNA binding domain, respectively, phosphorylation of these residues might be directly involved in the transcriptional regulation of *LCE1C* (Fig. 1h). For example, because ΔNp63 mutant harbors T281 in the DNA-binding domain but not T46 in the transactivation domain, it is possible that GAK cannot sufficiently promote the transcriptional activity of ΔNp63. We also found that LCE1C is inclined to form oligomers (Fig. 4a–c), and that its dimeric form preferentially interacts with PRMT5 (Fig. 4d,e). IF indicated that LCE1C and PRMT5 co-localize in the cytoplasm, although a part of them localizes at the nuclear foci (Fig. 5). Mass spectrometry identified importin α as an association partner of LCE1C (Fig. 6a,b), and their association *in vivo* was confirmed by Co-IP (Fig. 6d). We propose that LCE1C dimer in the cytoplasm is transported into the nucleus via the interaction with importin α and other factor(s), and then LCE1C interacts with and translocates PRMT5 from the nucleus to the cytoplasm, thereby preventing the nuclear function of PRMT5 (Fig. 6e). Namely, LCE1C sequesters PRMT5 in the cytoplasm. Therefore, in cancer cells with ΔNp63, the insufficient expression of LCE1C may be defective in inhibiting the oncogenic activity of PRMT5. Moreover, because GAK is overexpressed in cancer cells^16^, GAK may aberrantly promote the DNA-binding activity, but not the transcriptional activity, of ΔNp63 in cancer cells expressing ΔNp63, thereby preventing the transcription of *LCE1C* by TAp63 in a dominant negative manner. This suggests a possibility that disruption of the GAK_TAp63 axis causes tumorigenesis.

Mice lacking p63 derived from heterozygous crosses die within a day of birth, due to dehydration and maternal neglect, which suggests an essential role of p63 in epidermal development^25–28^. These p63-null mice, lacking all p63 isoforms, also display additional developmental defects, including truncated forelimbs, absence of hind limbs, and lack of stratified epidermis^25,26^. ΔNp63 knock-in mice in which the ΔNp63-specific exon is replaced by GFP also display abnormally developed stratified epidermis, consisting of isolated clusters of disordered epithelial cells^29^. Introduction of TAp63 and/or ΔNp63 isoform under the K5 promoter into p63−/− mice by *in vivo* genetic complementation showed greater patches of differentiated skin and clear reformation of a distinct basal membrane^30^. Moreover, TAp63-null mice do not show any apparent morphological defects, but rather demonstrate an essential role of TAp63 in a process of DNA damage-induced oocyte death^31^. Further characterization of TAp63-null mice showed that they develop obesity, insulin resistance, and glucose intolerance, suggesting a link between TAp63 and genes that regulate longevity^32^. However, these reports do not explain why p63 is essential for ectodermal differentiation during embryogenesis.

Our results here at least partially explain the abnormal phenotype of skin morphogenesis, since TAp63 controls transcription of *LCE1C* in a TAp63-T46/T281 phosphorylation-dependent manner (Figs 1–3). Removal of GAK from brain, liver, or skin using GAK conditional knockout mouse causes abnormal histology of these tissues, leading to the death of these mice shortly after birth^18^. MEFs derived from these GAK-null mice show deficiency not only in clathrin-mediated endocytosis due to the lack of clathrin-coated pits^19^, but also in proper cell division, ultimately becoming senescent^19^. These MEFs also harbor destabilized lysosomal membranes due to disruption of intracellular trafficking, thereby causing DNA damage because of iron leakage^19^. Mice expressing only the kinase-dead form of GAK (GAK-kd) die immediately after birth and display abnormal lung development, showing pulmonary alveolar dysfunction with abundant surfactant protein A within alveolar spaces^33^. We surmise that GAK-kd mice fail to conduct TAp63-pT46/pT281 phosphorylation, thereby leading to abnormal lung development possibly through insufficient expression of LCE1C, albeit the expression of TAp63 in epithelial cells is controversial. Although we do not exclude the possibility that other protein kinases also mediate TAp63-pT46/pT281 phosphorylation, similar phenotypes between p63-null and GAK-kd mice suggest that GAK plays a crucial role in TAp63-pT46/pT281 phosphorylation.

We here did not perform the detailed analysis on the C-terminus where there are multiple splice variants with very different transcriptional activities. The TAp63α isoform we used here is usually expressed in oocytes where it forms an inactive conformation^34,35^, and must be activated first through phosphorylation by checkpoint kinase 2 (CHK2)^36^. Because threonine rather than serine is favored as the phosphorylation site by GAK and no apparent consensus amino acid sequences are present around it^37,38^, effects of phosphorylation appears to be distinct from that of CHK2. Thus, it is our future research subject if phosphorylation TAp63-pT46/pT281 has any influence on female infertility in the oocytes. Taken together, we propose that the GAK_TAp63-pT46/pT281_LCE1C axis plays a role in normal mouse development.

## Materials and Methods

### Cell culture and preparation of Tet-ON advanced cells

Human osteosarcoma U2OS, human embryonic kidney (HEK) 293T, and human cervical cancer HeLa S3 cells, purchased from the American Type Culture Collection, were maintained in Dulbecco’s Modified Eagle’s Medium (DMEM; Sigma-Aldrich, D5796) supplemented with 10% fetal bovine serum (FBS) (HyClone, SV30014.03) with 100 U/mL penicillin and 100 μg/mL streptomycin (Nacalai Tesque, #26253-84). For preparation of Dox-inducible U2OS advanced cells, pTet-On advanced plasmid vector (Clontech) was transfected into cells. The cells were incubated in DMEM supplemented with 10% FBS, penicillin/streptomycin, and 0.2 mg/mL G418 disulfate aqueous solution (Nacalai Tesque, #16513-26), from which several single colonies were selected for preparation of Tet-ON inducible cell lines. For culture of pTet-On_advanced cells, 0.2 mg/mL G418 was added to the medium.

### Antibodies

Antibodies raised against the following proteins were purchased from the indicated commercial sources. Monoclonal antibodies: Myc-tag (clone PL14), Myc-tag HRP-DirecT, and GAPDH HRP-DirecT (MBL, Medical and biological laboratories co. Ltd.); p63 (CST, Cell Signaling Technology) and FLAG-tag and α-tubulin (SIGMA). Polyclonal antibodies: FLAG-tag (SIGMA); Myc-tag and α-tubulin HRP-DirecT (MBL); and PRMT5 and Importin-α/KPNA2 (CST). Anti-TAp63-pT281 (QYVEDPI[pT]GRQSVLC) and anti-LCE1C (TPKCPPKCPTPKCPP) polyclonal antibodies were constructed by Genscript. Non-stimulated A431 cell lysates (Millipore, #12-301) were used to check for the expression and band size (molecular weight) of endogenous ΔNp63 protein.

### DNA microarray and qRT-PCR analyses

DNA microarray and qRT-PCR analyses were performed as described previously^39^. The detailed microarray data have been deposited in the Gene Expression Omnibus (GEO; www.ncbi.nlm.nih.gov/geo) database (accession number GSE94635).

### Luciferase reporter assay

HEK293T cells were co-transfected with pGL3 firefly luciferase reporter vector (Promega) containing a series of *LCE1C* promoter region (region #1, #2, #3, or #4), pRL *Renilla* luciferase internal control vector (Promega), and pCMV6myc mammalian expression vector containing a series of TAp63 (6Myc-tagged TAp63-WT, -AA, or –DD) using PLUS Reagent and Lipofectamine Reagent (Invitrogen). After transfection, the cells were incubated for 48 h, and the luciferase activity was assayed using a Dual-Luciferase Reporter Assay System (Promega) according to the manufacture’s instruction. The results were normalized against *Renilla* luciferase activity. SV40 promoter was used as a positive control of luciferase assay. All data are shown as means ± s.d. (standard deviation) from three independent experiments.

## Electronic supplementary material

Supplementary Table S1

Supplementary information
