# Supplementary material for: Late cornified envelope 1C (LCE1C), a transcriptional target of TAp63 phosphorylated at T46/T281, interacts with PRMT5
Source: Sci Rep. 2018 Mar 20;8:4892. doi: 10.1038/s41598-018-23045-7 (PMC5861035; doi:10.1038/s41598-018-23045-7)
Supplement: Supplementary file 2 — Supplementary information [file 41598_2018_23045_MOESM2_ESM.pdf]

Supplementary information

Late cornified envelope 1C (LCE1C), a transcriptional target of TAp63 phosphorylated at T46/T281, interacts with PRMT5

Norikazu Yabuta, Chie Ota, Towa Sasakura, Yoko Naito, Daisuke Okuzaki,
Kohshiro Fukushima and Hiroshi Nojima

Materials and methods

Wb and dot blot analyses

For Wb analysis, cells were lysed in TNE250 buffer (10 mM Tris-HCl [pH8.0], 250 mM NaCl, 1 mM EDTA, 0.25% NP-40, and 2 mM benzamidine) including protease inhibitors (1 mM PMSF, 10 μg/mL leupeptin, 1 μg/mL aprotinin, 1 μg/mL pepstatine A, 1 mM DTT, 1 mM NaF, 1 mM Na_3VO_4, 10 mM β-glycerophosphate, and 100 ng/mL okadaic acid) at 4°C for 30 min. After centrifugation (4°C, 15,000 rpm, 30 min), supernatant was collected and the protein concentration was determined by the Bradford method. The supernatant was mixed with sample buffer (0.4 M Tris-HCl pH 6.8, 20% glycerol, 8% SDS, 10% 2-mercaptoethanol, and 0.2% bromophenol blue), and boiled. After SDS-PAGE, transfer to PVDF membrane (Millipore) was performed. For dot blot analysis, antigenic peptides were loaded onto PVDF Immobilon-P transfer membrane (Millipore, Bedford, MA). Blocking was performed by TBST containing 5% nonfat skim milk or 5% BSA. Antibody-specific dots or bands were visualized by Lightning Chemiluminescence Reagent Plus (Perkin-Elmer).

Preparation of Tet-ON inducible cell lines

The pTRET3-6Myc vector was constructed by inserting a multiple cloning site linker containing 6Myc-tag (*Bam*HI-*Hin*dIII-*Cla*I-6Myc-*Asc*I-*Eco*RV-*Not*I-*Sal*I) into *Bam*HI and *Sal*I sites of pTRE-Tight vector (Clontech). U2OS/Tet-On cells were co-transfected with pTRET3-6Myc plasmids (Myc-vector, TAp63-WT, TAp63-pT46A/pT281A, or TAp63-pT46D/pT281D) and linear hygromycin marker (Clontech) using lipofectamine and PLUS reagent according to the manufacturer’s instructions (Invitrogen). Transfected cells were diluted and selected with selection medium including hygromycin (200 μg/mL). Single colonies were picked up and positive clones were identified by Wb analysis after 48 h incubation in the presence or absence of doxycycline (Dox; 1 μg/mL).

Liquid chromatography-tandem mass spectrometry (LC-MS/MS)

For mass spectrometric analysis, U2OS cells were transfected with FLAG-tagged plasmid DNA harboring human FLAG-LCE1C or FLAG-vector and the cell extracts were immunoprecipitated with anti-FLAG antibody and protein A-Sepharose. After washing with NETN150 (20 mM Tris-Cl [pH 8.0], 150 mM NaCl, 1 mM EDTA and 0.5% Nonidet P-40], the denatured precipitates were separated by SDS-PAGE and stained with 2D-SILVER STAIN-II (Cosmo Bio Co., LTD, Tokyo, Japan). The indicated bands were cut out, subjected to in-gel trypsin protease digestion, and the peptides were analyzed by nanocapillary reversed-phase LC-MS/MS using a C18 column (f 0.1 × 150 mm) on a nanoLC system (Advance, Michrom BioResources, Auburn, CA) coupled to an LTQ Orbitrap Velos mass spectrometer (Thermo Fisher Scientific, Waltham, MA). To identify proteins, database searching was performed in-house using the MASCOT Server (Matrix Science, London, UK).

Purification of recombinant proteins

Each p63 fragment was inserted into the *Asc*I and *Not*I site of pGEX6p (+*Asc*I) vector and introduced into *E. coli* BL21 (RIL). The cells were then collected and lysed in phosphate buffered saline (PB containing 1% Triton X-100, 1 µg/mL leupeptin, 1 µg/mL aprotinin, 1 µg/mL pepstatin A, 1 mM benzamidine, 100 µg/mL PMSF, 1 mM NaF, and 1 mM Na_3VO_4 by brief sonication. After centrifugation, the clear lysate was adsorbed to Glutathione Sepharose 4B (Amersham Pharmacia Biotech, #27-4574-01). Then, GST-fused protein was eluted from the column by elution buffer containing 10 mM reduced glutathione (Nacalai Tesque, #08786-61).

Kinase assays in vitro

GST-fused p63 fragments that were prepared as purified recombinant proteins were used as substrates. For *in vitro* kinase assays, 0.8 µg of GST-purified rat-GAK-k and GST-purified substrates were mixed into the kinase buffer [10 mM HEPES (pH 7.5), 50 mM NaCl, 10 mM $MgCl_2$, 5 mM $MnCl_2$, 1 mM DTT, 5 mM NaF, and 50 mM β-glycerophosphate] including [γ-^{32}P]-labeled 5 µM ATP (10 µCi, Perkin-Elmer, NEG502A). Each reaction mixture was incubated at 30°C for 30 min, and the reaction was stopped by addition of Laemmli buffer. Samples were boiled for 5 min before analysis by SDS-PAGE, and stained with Simply Blue^{TM} Safe Stain (Invitrogen). The [γ-^{32}P] signals were detected by image analyzer FLA7000 (Fujifilm). In the non-radioactive assay, non-labeled ATP was used and detected by phospho-specific antibodies.

Plasmid transfection and IP

HEK293T or U2OS cells were transfected with a pCMV-FLAG-vector, into which the relevant DNA fragments were inserted. Transfection was performed using PLUS Reagent and

Lipofectamine Reagent (Invitrogen). After 48 h, cells were lysed in TNE250 buffer containing inhibitors (10 mM Tris-HCl [pH8.0], 250 mM NaCl, 1 mM EDTA, 0.25% NP-40, 2 mM benzamidine, 100 μg/mL PMSF, 10 μg/mL leupeptin, 1 μg/mL aprotinin, 1 μg/mL pepstatine A, and 0.1 μM okadaic acid), and the protein concentration of the lysate was determined by the Bradford method. For each IP, 2 mg cell extract was used (0.1 mg for Input).

Indirect IF analyses

HeLa S3 or U2OS cells were plated on coverslips and fixed by sequential incubations at room temperature in 4% formaldehyde in PBS without Ca^{2+} and Mg^{2+}, PBS(-), and 0.1% Triton X-100 in PBS(-), then 0.05% Tween-20 in PBS(-), each for 10 min. Cells were fixed at -20°C in methanol for 10 min and washed with PBS(-). Blocking was performed by TBST (200 mM Tris-Cl [pH 7.5], 150 mM NaCl, and 0.05% Tween-20] containing 5% FBS. Cells were incubated with relevant antibodies, followed by incubation with anti-rabbit/mouse IgG conjugated with AlexaFluor 488 and 594 (Molecular Probes) in TBST containing 5% FBS. DNA was stained with Hoechst 33258 (Sigma, St. Louis, MO). The stained cells were observed with a confocal laser scanning microscope (Olympus, Fluoview_FV10i). IF images were acquired using Photoshop (Adobe).

Statistical analysis

Significant differences were determined by Student's t-test. The data are expressed as means ± SE. $P < 0.01$ was considered to be statistically significant.

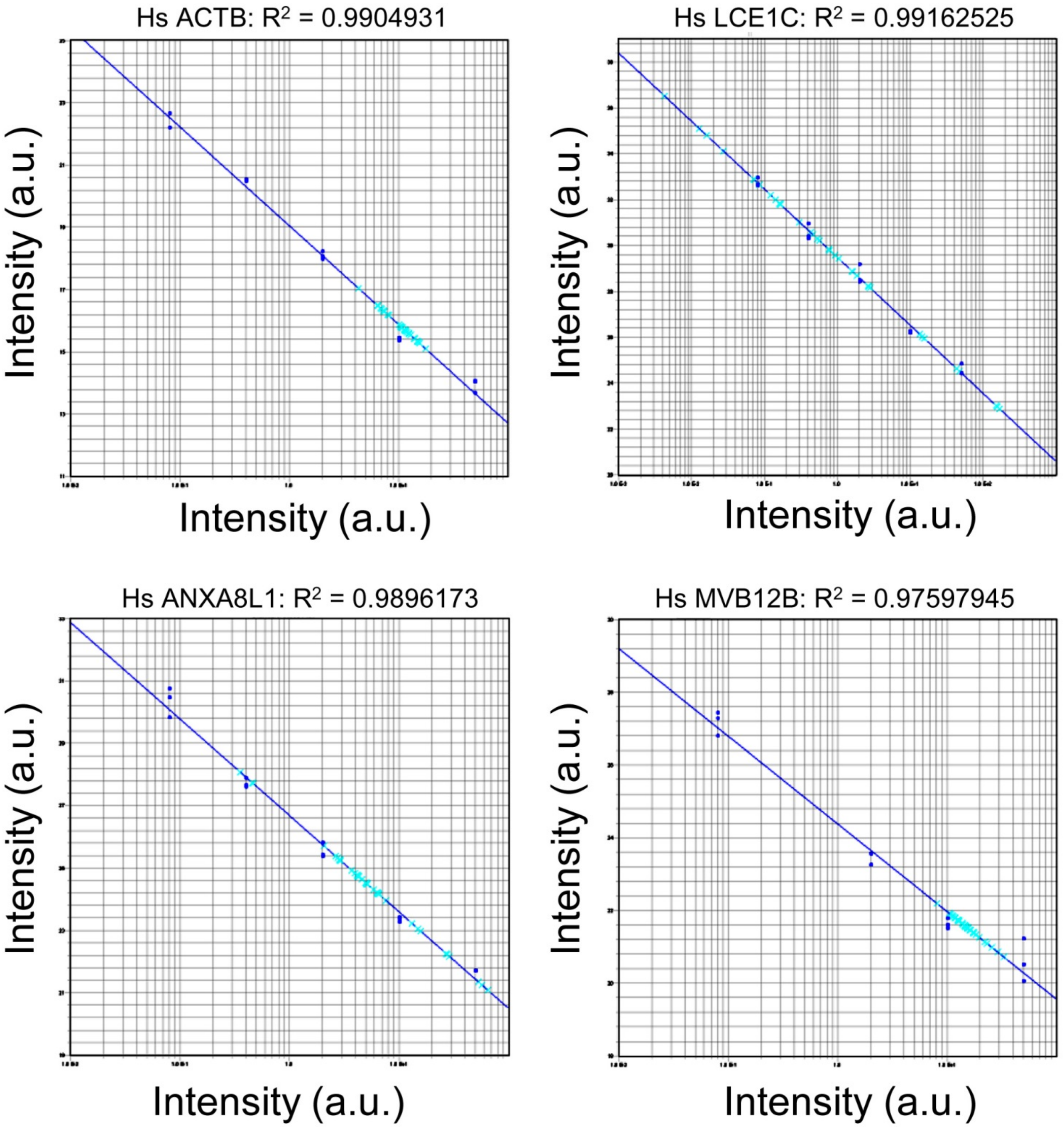

Figure S1. Standard curve of qRT-PCR used for indicated genes. Linear distribution of the dots derived from the data suggests a successful qRT-PCR experiment. ACTB encodes β-actin protein.

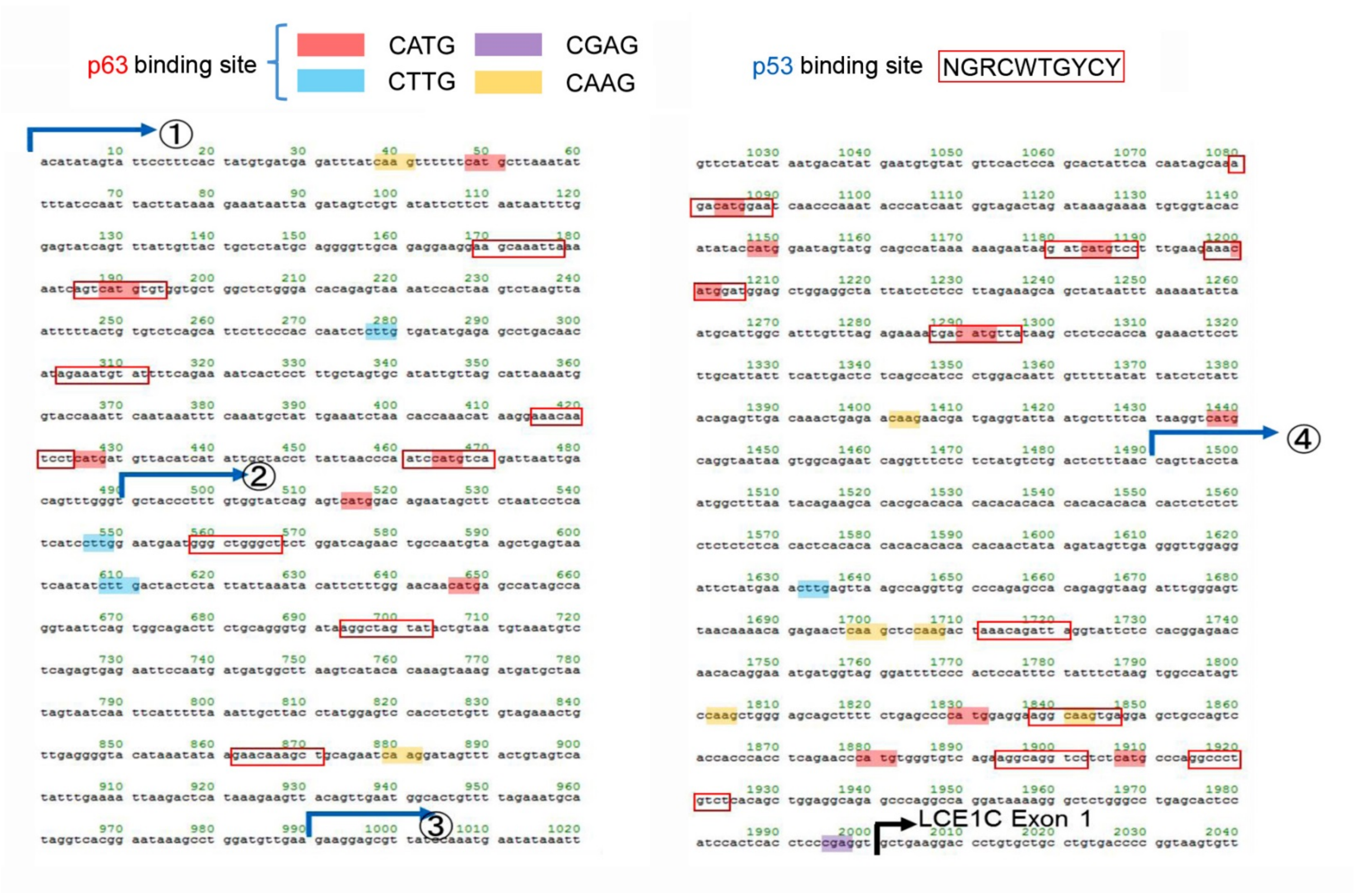

Figure S2. DNA sequence in the promoter region of the LCE1C gene. Putative DNA sequences for p53 and/or p63 binding sites are boxed by red lines or denoted by colored shading. The locations of deletion fragments (#1–4) are indicated by blue arrows.

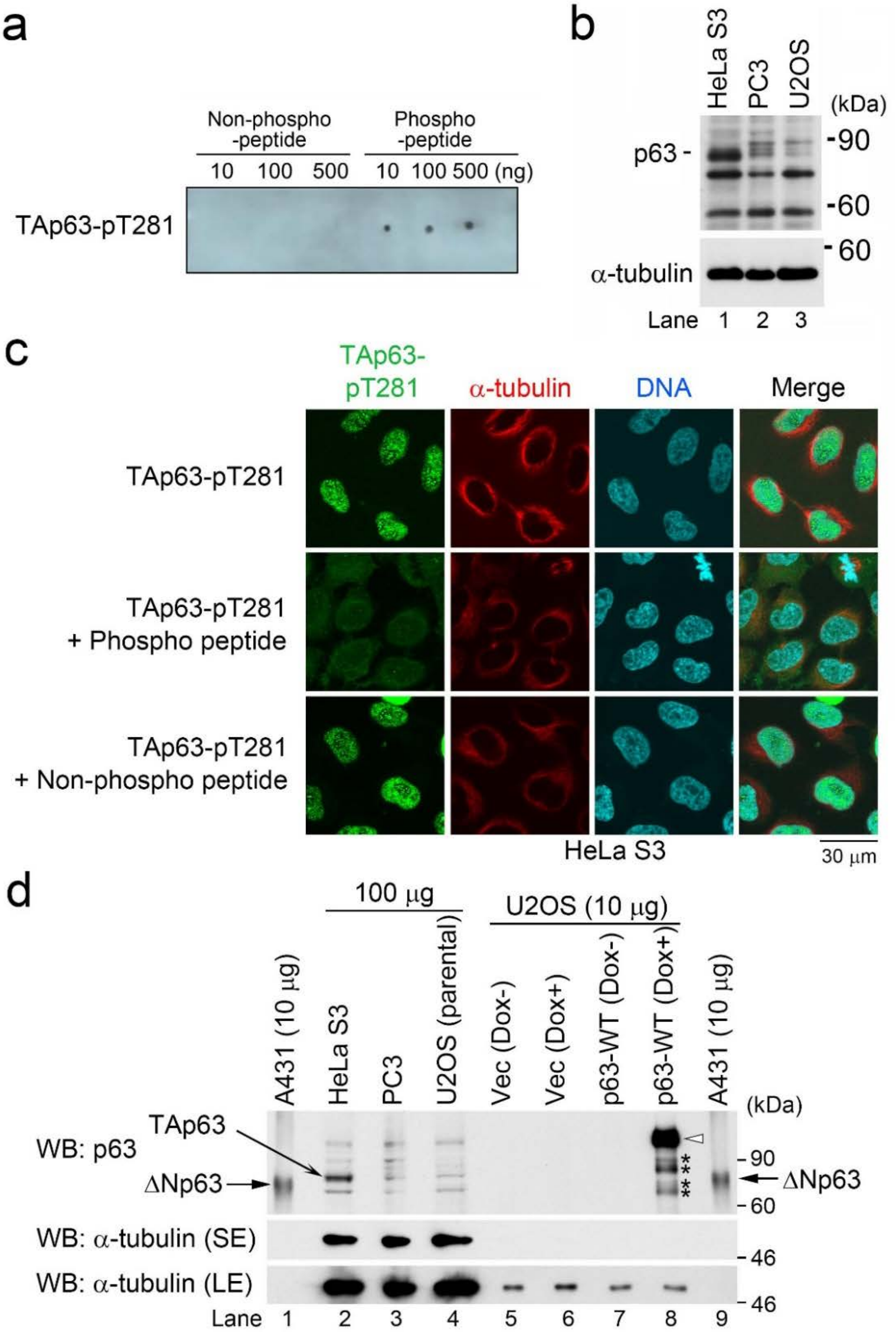

Figure S3. Quality check of the anti-TAp63-pT281 antibody. **(a)** Peptide dot blot analysis of the anti-TAp63-pT281 polyclonal antibody generated using the KLH-conjugated phosphopeptide (QYVEDPI[pT]GRQSVLC) and non-phosphopeptide (QYVEDPITGRQSVLC). These antibodies recognized the phosphopeptides, but not non-phosphopeptide, at indicated peptide concentrations. **(b)** Wb identified p63 expression in the extract from HeLa S3 and PC3 (a prostate cancer cell line) cells. U2OS cells had little or no expression of p63. α-tubulin is a loading control. **(c)** Peptide competition assay for IF using anti-TAp63-pT281 polyclonal antibody in the presence of phosphopeptide or non-phosphopeptide. IF signals (green) detected by anti-TAp63-pT281 antibody almost disappeared by addition (+) of phosphopeptide, but not non-phosphopeptide, suggesting a successful peptide competition experiment. Bar, 30 μm. **(d)** Endogenous ΔNp63 is not detected in parental U2OS cells and U2OS cell lines expressing 6Myc-TAp63-WT. Open arrowhead and Asterisks show 6Myc-tagged TAp63-WT and its degraded products, respectively. A431 and HeLa S3 cells were used as control cells expressing endogenous ΔNp63 and TAp63, respectively. α-tubulin is a loading control (SE, short exposure; LE, long exposure) , which is very low or undetectable expression levels in A431 cells (10 μg) compared with U2OS cells (10 μg).

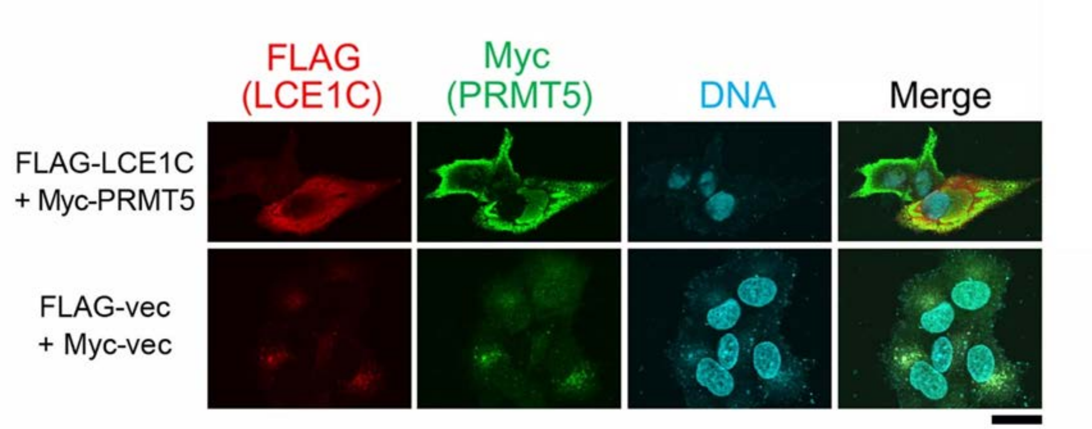

Figure S4. Subcellular localization of FLAG-LCE1C and Myc-PRMT5 in U2OS cells expressing these proteins. Blue bar, 30 μm.

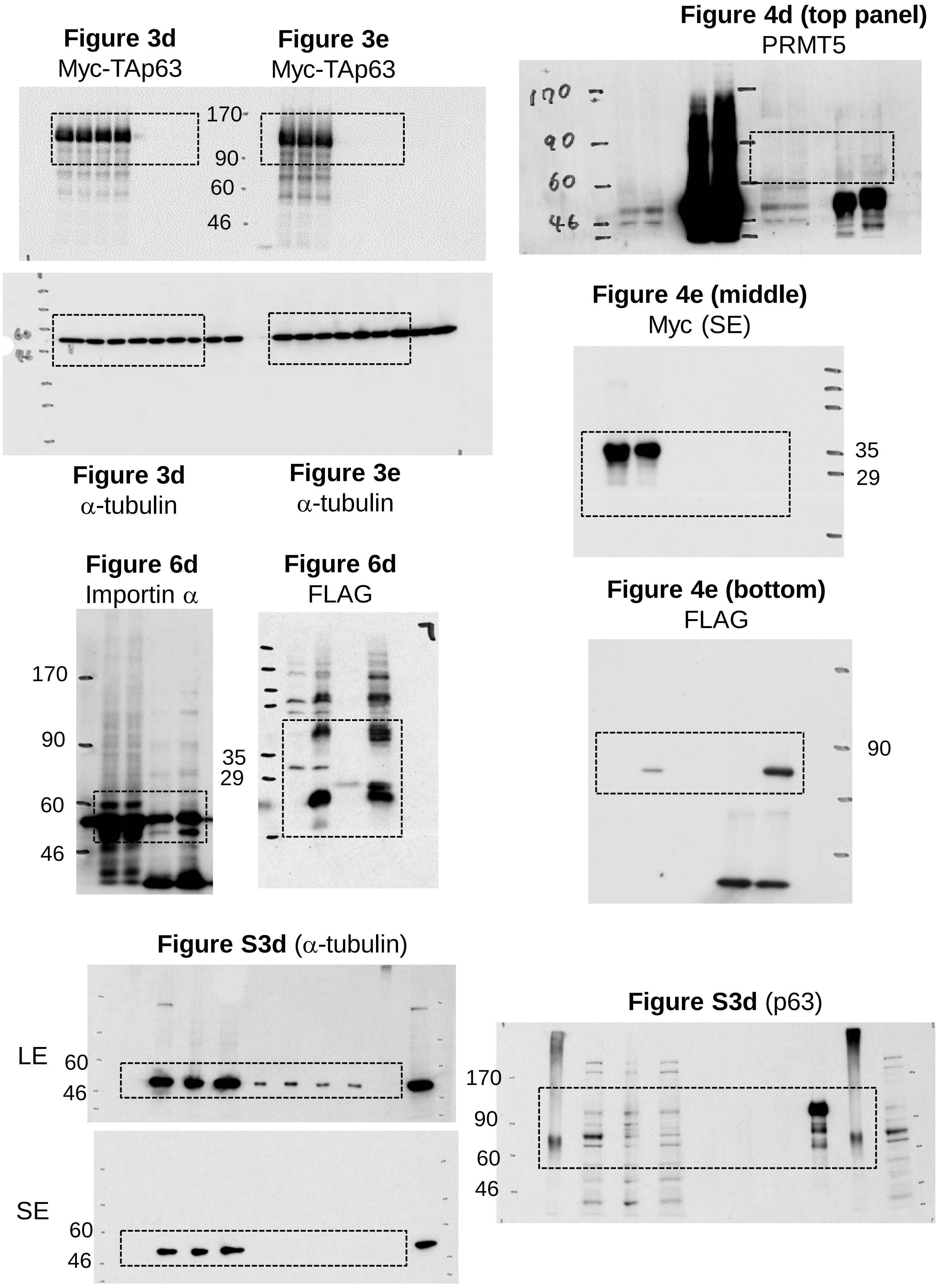

Figure S5. Full scan images of Figures 3d, 3e, 4d (top panel), 4e (middle and bottom panels), 6d, and S3d.
